# Assessment of atrial septal defects in adults comparing cardiovascular magnetic resonance with transoesophageal echocardiography

**DOI:** 10.1186/1532-429X-12-44

**Published:** 2010-07-22

**Authors:** Karen SL Teo, Patrick J Disney, Benjamin K Dundon, Matthew I Worthley, Michael A Brown, Prashanthan Sanders, Stephen G Worthley

**Affiliations:** 1 Cardiovascular Research Centre, Royal Adelaide Hospital and The University of Adelaide, Adelaide, South Australia

## Abstract

**Background:**

Many adult patients with secundum-type atrial septal defects (ASDs) are able to have these defects fixed percutaneously. Traditionally, this has involved an assessment of ASD size, geometry and atrial septal margins by transoesophageal echocardiography (TOE) prior to percutaneous closure. This is a semi-invasive technique, and all of the information obtained could potentially be obtained by non-invasive cardiovascular magnetic resonance (CMR). We compared the assessment of ASDs in consecutive patients being considered for percutaneous ASD closure using CMR and TOE.

**Methods:**

Consecutive patients with ASDs diagnosed on transthoracic echocardiography (TTE) were invited to undergo both CMR and TOE. Assessment of atrial septal margins, maximal and minimal defect dimensions was performed with both techniques. Analyses between CMR and TOE were made using simple linear regression and Bland Altman Analyses.

**Results:**

Total CMR scan time was 20 minutes, and comparable to the TOE examination time. A total of 20 patients (M:F = 5:15, mean age 42.8 years ± 15.7) were included in the analyses. There was an excellent agreement between CMR and TOE for estimation of maximum defect size (R = 0.87). The anterior inferior, anterior superior and posterior inferior margins could be assessed in all patients with CMR. The posterior superior margin could not be assessed in only one patient. Furthermore, in 1 patient in whom TOE was unable to be performed, CMR was used to successfully direct percutaneous ASD closure.

**Conclusions:**

CMR agrees with TOE assessment of ASDs in the work-up for percutaneous closure. Potentially CMR could be used instead of TOE for this purpose.

## Background

Atrial septal defects are the most common congenital cardiac malformation first diagnosed in adults and account for approximately 10% of all congenital heart lesions [1]. Patients with a significant shunt (Qp/Qs > 1.5/1.0) experience symptoms over time with effort dyspnoea seen in about 30% of patients by the third decade and in over 75% of patients by the 5^th ^decade. Complications may include the development of pulmonary hypertension, supraventricular arrhythmias (atrial fibrillation and atrial flutter) and right-sided heart failure from right ventricular volume overload.

Surgical closure of atrial septal defects (ASDs) has previously been shown to have excellent results in both medium and long term studies [2], but is associated with significant morbidity and mortality [3]. Many adults with secundum ASDs are now able to have these defects closed percutaneously using septal occluder devices such as the Amplatzer Septal Occluder (ASO), a self-expanding circular double disc with a conjoint waist containing polytetrafluoroethylene (PTFE) and a nitinol mesh. This device has now become an accepted alternative to surgical repair with studies comparing ASO device closure to surgical closure showing decreased complication rates, shorter hospital stays and greater cost-effectiveness [4].

The echocardiographic morphology of the ASD and accurate assessment of the stretched diameter has been important for patient selection. Initially, it was felt that atrial septal defects up to 26 mm stretched balloon diameter could be closed with the ASO [5]. More recently, there is registry data of larger ASDs closed successfully using the 40 mm ASO [6]. Inclusion criteria for percutaneous ASD closure have included: 1) the presence of a secundum ASD <40 mm by echocardiography, 2) a left-to-right shunt with a Qp/Qs ratio of >1.5:1 or the presence of right ventricular volume overload 3) patients with minimal shunt in the presence of symptoms and 4) the presence of a distance of >5 mm from the margins of the ASD to the coronary sinus, atrioventricular valves and right upper pulmonary vein as measured by echocardiography [7-9]. Traditionally, these assessments of ASD size, geometry and atrial septal margins have been obtained by transoesophageal echocardiography (TOE) prior to the percutaneous closure [7,10]. This is a semi-invasive technique and all of the information could potentially be obtained by non-invasive cardiovascular magnetic resonance (CMR). Furthermore, TOE is imperfect at assessing all atrial septal margins. A large atrial septal defect that is located inferoposteriorly is difficult to both assess and close [7] and relates to the limited assessment of the posterior inferior margin by TOE [11]. Although 3-D TOE may improve this, this new technology is not yet widely available, and yet to be shown to be superior to 2-D TOE for this purpose.

The aim of this study was to compare the assessment of atrial septal defects in consecutive adult patients being considered for percutaneous ASD closure using CMR and TOE.

## Methods

### Subjects

Consecutive patients with secundum atrial septal defects diagnosed on transthoracic echocardiography (TTE) were invited to undergo both transoesophageal echocardiography (TOE) and CMR.

### Transoesophageal echocardiographic imaging

TOE was performed with a Sonos 5500 (Phillips) TOE system with a multiplanar 7.0mHz phased-array transducer. Cross-sectional studies of the atrial septum were performed utilising multi-plane views as previously described: mid-oesophageal 4-chamber views, short axis view and biatrial long axis views [7].

### Cardiovascular Magnetic Resonance

CMR studies were performed with subjects in the supine position using a 1.5 Tesla scanner (Siemens Sonata, Germany) and a phased array surface coil. Images were obtained during end-expiratory breath-hold (8 to 10 seconds) with retrospectively cardiac-gated True FISP (Fast imaging with steady-state free precession) sequences. Both short (modified bi-atrial) and long axis images (4 chamber views) were obtained through the ASD, with section thickness of 6 mm and no intersection gap. Thus, consecutive slices were obtained to cover the whole of the interatrial septum in both short and long axis views. Assessment of atrial septal margins, maximal and minimal defect dimensions in both short and long axis views was performed.

### Measurement of atrial septal margins and defect size

**Figure 1:**
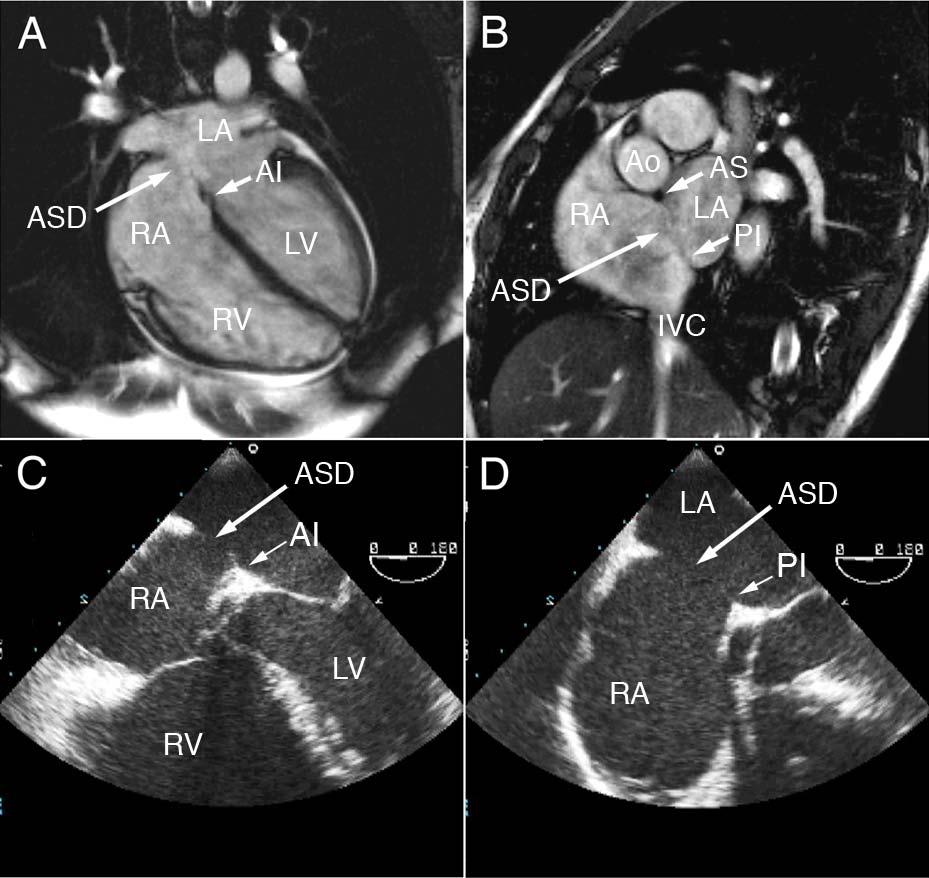
**Atrial septal defect (ASD) and margins imaged with CMR (panel A - 4 chamber view, panel B - modified biatrial short axis view) and transoesophageal echocardiography (panel C - 4 chamber view, panel D - biatrial short axis view)**. Margins are denoted with AS anterior superior rim, AI anterior inferior, PS posterior superior, PI posterior inferior.

TOE and CMR scans were reviewed retrospectively and 2 independent observers performed the measurements. Images were reviewed for (a) assessibility of defect size and septal margins by TOE, and (b) assessibility of defect size and septal margins by CMR, (c) Agreement of the measurements between TOE and CMR. The maximal diameters of the atrial septal defects were measured on both TOE and CMR. Atrial septal margins were measured as previously published [11,12]. The anterior inferior (AI) rim was measured from the defect to the mitral valve, the anterior superior (AS) rim from the defect to the aortic root, posterior inferior (PI) rim from the defect to the inferior vena cava and posterior superior (PS) rim from the defect to the superior vena cava (see Figure 1).

The TOE views for measurement of the rims were: the mid-oesophageal four-chamber view for AI rim, basal short axis view for AS rim and biatrial views for PI and PS rims. Corresponding CMR views were the four-chamber view for AI rim, short axis view through the aorta for the AS rim and modified biatrial short axis views for PS and PI measurements. The size of the device occluder device used was also compared to the maximal defect size on CMR and TOE in patients who subsequently underwent ASD closure.

### ASD device implantation

All implantation of the ASD devices with the Amplatzer Septal occluder were performed with TOE guidance and fluoroscopy. A sizing balloon was used to determine the stretched diameter of the ASD before selection and deployment of ASO device, as previously described [8]. In brief, a Meditech balloon (Boston Scientific, Watertown, MA) sized 20 or 27 mm was used in this series. This balloon was inflated within the left atrium and firm continuous pressure applied to pull it into the atrial septum, using TOE guidance. The diameter at which the balloon just gets through the atrial septal defect was the stretched balloon diameter (SBD).

### Statistical Analysis

Data is presented as mean ± standard deviation. Analyses between TOE and CMR were made using simple linear regression analysis (SPSS 11.0 software) and Bland Altman analysis [13]. Statistical significance was taken at a p value of 0.05.

## Results

A total of 20 patients (M: F = 5: 15, mean age 42.8 years ± 15.7) were included in the analyses. All but 1 patient had both TOE and CMR to evaluate their suitability for percutaneous ASD closure. TOE was not performed in 1 patient due to history of an oesophageal stricture. CMR was well tolerated in all patients. Total CMR examination time was 20 to 30 minutes in all cases. Three patients were found not to be suitable for percutaneous ASD closure due to excessive stretched balloon diameter (SBD) of >40 mm at cardiac catheterisation (CMR diameters pre-balloon sizing were 32, 38 and 33 mm). All 3 patients went on to surgical closure.

### Assessibility of defect and margins with TOE

The maximal defect size could be assessed in all patients (n = 19) with TOE. The anterior inferior (AI) margin, which was measured from the defect to the mitral valve in the mid-oesophageal 4-chamber view could be assessed in all patients. The anterior superior margin was assessable in 79%, the posterior inferior margin in 63% and the posterior superior margin in 74% of patients (See Table 1).

**Table 1:** Assessment of atrial septal defect size and margins (CMR vs. TOE)

	Able to be assessed by CMR (%)	Able to be assessed by TOE (%)
Maximal defect size	(20/20) 100%	(19/19) 100%
Anterior superior margin	(20/20) 100%	(15/19) 79%
Anterior inferior margin	(20/20) 100%	(17/19) 89%
Posterior superior margin	(19/20) 95%	(14/19) 74%
Posterior inferior margin	(20/20) 100%	(12/19) 63%

### Assessibility of defect and margins with CMR

The maximal defect size could be assessed in all patients (n = 20) with CMR. The anterior inferior, anterior superior and posterior inferior margins could be assessed in all patients. The posterior superior margin could not be assessed in only one patient.

### Agreement between TOE and CMR

There was a good agreement between CMR and TOE for estimation of maximum defect size (R = 0.87) and minimum defect size (0.92). There was similar agreement between CMR and TOE versus size of the ASO device (Maximum defect size by CMR vs. ASO size, R = 0.53 and maximum defect size by TOE vs. ASO size R = 0.57). See Table 2.

**Table 2:** Maximal ASD diameters and Amplatzer septal occluder size

Patient	Maximal MRI Diameter (mm)	Maximal TOE Diameter (mm)	ASO Size (mm)
1	19.7	16	26
2	11.8	11	18
3	25.2	21	32
4	17.9	21	32
5	19.1	23	20
6	15.1	14	18
7	12.7	14	24
8	25.9	35	38
9	19.2	18	26
10	15.7	12	20
11	11.4	10	16
12	15.9	19	30
13	20	21	26
14	24.2	Not Performed	30
15	23.6	22	28
16	25	27	28
17	9.2	10	20

**Figure 2:**
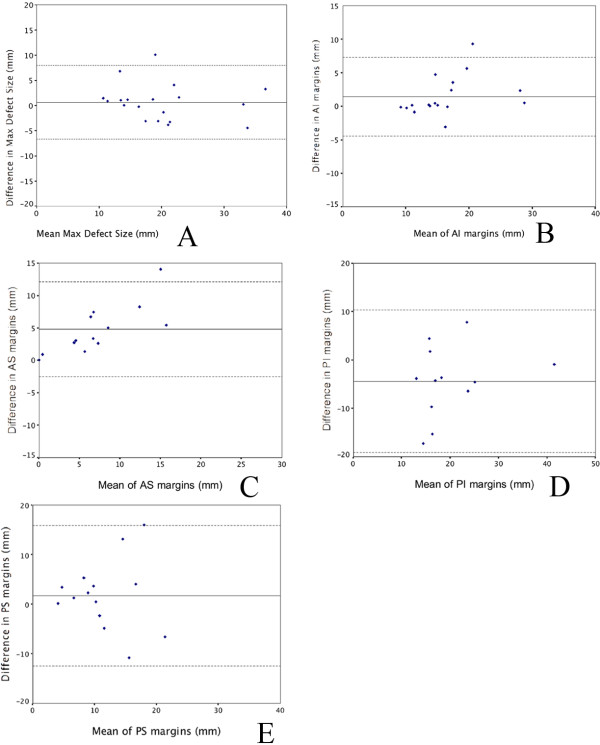
**Bland Altman comparative analyses of the mean ASD dimensions and difference in dimensions between CMR and TOE. (A) **Maximum defect size, **(B) **Anterior inferior (AI) margins, **(C) **Anterior superior (AS) margins, **(D) **Posterior inferior (PI) margins, and **(E) **Posterior superior (PS) margins.

Bland and Altman analyses comparing CMR and TOE measurements of defect size and atrial septal margins are shown in Figures 2A to E and are presented as mean of the 2 measurements obtained by CMR and TOE ± difference between the 2 measurements.

## Discussion

Many adults with secundum atrial septal defects are now able to have these defects closed percutaneously. The size, location and margins of an atrial septal defect are major determining factors for transcatheter closure. Conventional assessment pre-closure have been with transoesophageal echocardiography (TOE) for measurement of ASD dimensions and margins[7,10]. Inclusion criteria for closure have included ASD maximum diameter of 40 mm and rims of at least 5 mm towards the IVC, SVC, right upper pulmonary vein and mitral valve [8,9]. However, more recently, deficiency of the margins has still allowed closure. For example, in patients with a deficient anterior superior (AS) margin, a correctly sized Amplatzer septal occluder can successfully be used with the device moulding itself around the aortic wall with minimal risk of perforation [8,9]. There have also been small numbers of patients with small inferior and posterior defect margins (<5 mm) who have been successfully closed [9].

While TOE has been the traditional method for the evaluation and screening for patients who are candidates for transcatheter closure, it is semi-invasive and cannot easily be performed in young children and some adults. Measurements of the ASD can only be obtained from the 4-chamber, short axis and bicaval views on TOE due to the plane on TOE that generally projects the ASD in a relatively fixed direction [11]. In addition, TOE may underestimate the size of the stretched balloon diameter of the ASD, although this has been described to factors associated with choice of plane for maximal diameter in defects that are oval rather than circular in shape, displacement of the interatrial septum from enlarged right atrium and changes of the shape of the defect during the cardiac cycle [7,14]. Three-dimensional echocardiography obtained from TOE images may provide superior anatomic detail [10,15], but the accuracy of the reconstructed images is dependent on technical experience of the operator [8].

CMR has been shown to visualise secundum ASDs [16]. Studies comparing CMR with TOE to assess ASD and suitability for percutaneous closure have mainly been done in paediatric populations [11], although there are a limited number of studies with adult patients [17-19]

In this study, we were able to assess maximal atrial defect size in all patients with both TOE and CMR. In the assessment of atrial septal margins, CMR could assess the posterior inferior margins in all patients compared to approximately 60% that could be assessed with TOE. One study in a paediatric population comparing TOE and MRI assessment showed that patients who had successful closure had a significantly smaller major axis of ASD and larger posterior inferior rim compared to those who were excluded from closure procedure. An adequate posterior inferior rim was also best visualised in that study with CMR and showed a better correlation of ASD diameter measurement to balloon sizing compared to TOE [11]. However, a limitation in our study of the measurement of margins using TOE relates to this study being a retrospective study and with the images that were strictly required for measurement of the margins (such as the PI margin) not being available in some patients. Hence the 60% assessibility for the PI margin is not a true reflection of the ability of TOE to define this margin.

In the patients (n = 3) that went on to surgical closure of their defects, although the pre-procedure ASD diameters on CMR were less than 40 mm (between 32-38 mm in fact), the stretched balloon diameters were >40 mm, which is beyond the largest ASD amplatzer closure devices available, at the time of invasive assessment.

Velocity encoded contrast cine imaging was not performed in this study, however, this is another CMR technique that can provide information about the size and shape of the atrial septal defect [16,18] as well as provide accurate assessment of shunt magnitude from measuring flow in the systemic and pulmonary circulations [20,21]. However, this technique has not been used for the assessment of margins due to its limited spatial resolution.

## Conclusions

CMR agrees closely with TOE assessment of atrial septal defects for percutaneous closure. In addition, it is able to assess the septal margins such as the posterior inferior margin, which is known to be difficult to assess with TOE. Potentially, CMR could be used instead of TOE for the assessment for percutaneous closure.

## Competing interests

The authors declare that they have no competing interests.

## Authors' contributions

KSLT participated in the study design, CMR data acquisition, analysis and manuscript preparation. PJD and BKD participated in TOE data analysis and manuscript revision. MAB, MIW and PS contributed to manuscript revision. SGW conceived the study and revised the manuscript. All authors read and approved the final manuscript.
